# Pharmacy Students’ Attitudes and Perceptions of “Virtual Worlds” as an Instructional Tool for Clinical Pharmacy Teaching

**DOI:** 10.3390/pharmacy5010005

**Published:** 2017-02-05

**Authors:** Claire Englund, Maria Gustafsson, Gisselle Gallego

**Affiliations:** 1 Center for Educational Development (UPL), Umeå University, SE-90187 Umeå, Sweden; claire.englund@umu.se; 2 Department of Pharmacology and Clinical Neuroscience, Umeå University, SE-90187 Umeå, Sweden; gisselle.gallego@umu.se; 3 School of Medicine, The University of Notre Dame, Australia, New South Wales 2010, Australia

**Keywords:** three-dimensional virtual worlds, clinical pharmacy teaching, pharmacy school

## Abstract

The objectives of this study were to explore pharmacy students’ perceptions and experiences of three-dimensional virtual worlds (3DVWs) as an instructional tool for clinical pharmacy teaching. Semi-structured interviews were carried out with Master of Science in Pharmacy students who had participated in communicative exercises in a 3DVW. Interviews were digitally recorded, transcribed and analyzed using thematic analysis. More than half of the students were positive to using 3DVWs for educational purposes and see the advantages of having a setting where communication can be practiced in an authentic but ‘safe’ environment available online. However, many students also reported technical difficulties in using the 3DVW which impacted negatively on the learning experience. Perceived ease of use and usefulness of 3DVWs appears to play an important role for students. The students’ level of engagement relates to not only their computer skills, but also to the value they place on 3DVWs as an instructional tool.

## 1. Introduction

Three-dimensional virtual worlds (3DVWs), such as OpenSimulator (http://opensimulator.org/) (OS) or SecondLife (SL), are online, multi-user three-dimensional computer-generated environments that simulate the real world. A 3DVW, also known as an immersive virtual world (IVW), is a simulated multimedia environment that provides graphical representation of a physical space where users can interact and communicate by voice chat through their own graphical and digital self-representation known as an ‘avatar’ [1,2]. The avatar is navigated by the user and may or may not resemble the persons’ appearance. 3DVWs can offer a wide range of learning opportunities, but also require a new way of thinking for both teachers and students [3]. The three-dimensional features of 3DVWs have the potential to create an engaging and realistic learning experience [4,5]. According to Savin-Baden 3DVWs have great educational potential in the form of role-playing and foster dialogical learning and social interaction [6]. There is an increasing interest in conducting teaching in 3DVWs, which can contribute to greater variety in the design of learning opportunities offered to students. Lim for example, described the “six learnings of Second Life” [7], including collaborative learning, where students work in teams to solve problems. According to Salmon [8], 3DVWs can aid collaborative learning in the form of cooperation and exchange, i.e., to exchange views and learn from each other to achieve a common goal.

As described by Ghanbarzadeh et al. [2], 3DVWs are an emerging method used in both traditional classrooms and distance education and have been utilized in disciplines such as healthcare education [9,10], teacher education [11,12], and language development [13]. There are however challenges to creating effective learning scenarios in 3DVWs, including technical issues such as bandwidth and the development of basic in-world competencies such as the need to manipulate and manage avatars in the 3DVW [11,14]. Despite its growing popularity in medicine and nursing, the uptake in pharmacy has been slow and most studies describing their use have been based in New Zealand, the United States (US), and the United Kingdom (UK) [2,15,16]. Some examples include but are not limited to: an elective course on drug safety for second and third year Doctor in Pharmacy students [17], an advanced therapeutics course which supplements lecture content [18], and an online virtual patient program to train pharmacy students and pharmacists in providing medication therapy management [19]. However, most of these courses are electives and limited information is available on established non-elective courses where students are required to participate in 3DVW learning activities to complete course objectives.

### 1.1. Education Environment

In response to the shortage of pharmacists in sparsely populated areas, Umeå University established its distance Bachelor of Science in Pharmacy program in 2003. In Sweden, there are two different pharmacy degrees: prescriptionist (“receptarie” in Swedish) and pharmacist (“apotekare” in Swedish). Prescriptionists have a Bachelor’s degree (three years) and most often work in community pharmacies. Pharmacists have a Master’s degree (five years) and are more involved with specialized tasks such as educational activities, medication reviews, research and development, and drug and therapeutics committees [20]. Umeå University offers a three year web-based Bachelor’s program [21], a supplementary two year web-based Master’s degree, and most recently a five year web-based Master of Science in Pharmacy program. Students from Sweden, Norway, and Finland are allowed to apply to all programs. However, they need to be able to understand and speak Swedish as all course materials and exercises are conducted in Swedish. Prescriptionists are also recognized in these countries but the role does not exist elsewhere. While face to face meetings are held on campus on a number of occasions, most of the teaching takes place online. Neither program is available as a campus-based option at Umeå University. The mode of delivery for all pharmacy programs at Umeå University is web-based.

**Figure 1:**
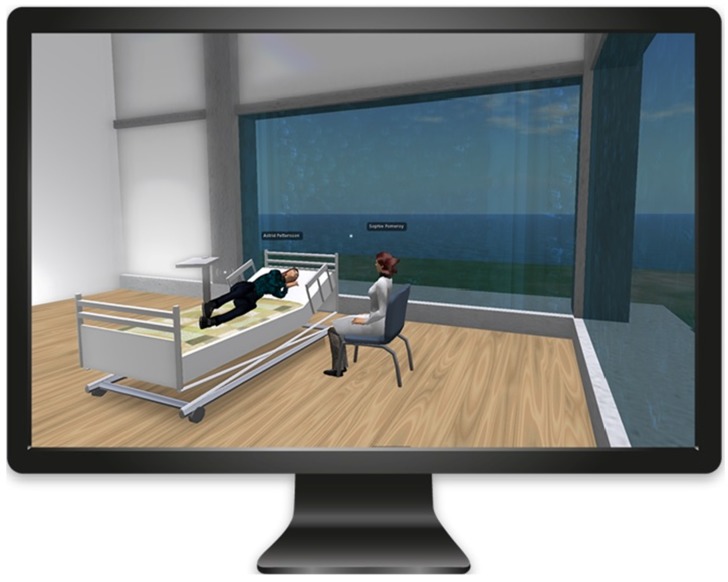
View of the virtual hospital ward where the pharmacist and the patient can meet and discuss the patient’s drug treatment.

In 2009, a virtual community pharmacy and later a hospital (see Figure 1) were developed in collaboration between the Center for Educational Development (UPL) and the pharmacy program at Umeå University. The aim was to develop a new pedagogical model to improve student learning and provide authentic opportunities for communication training for distance students through the development of virtual hospitals and community pharmacies. The 3DVW has been used in the clinical pharmacy course since its inception in 2011. In the clinical pharmacy course, students are provided with opportunities for training in simulated ward rounds and patient meetings in the 3DVW, where students can practice communication with patients and doctors in a professional manner. The virtual hospital environment was designed by the course teacher and built by an educational technologist from UPL using a three-dimensional modeling tool. The aim was to create a realistic setting, promoting a sense of immersion or ‘presence’ in the virtual environment. Childs [22] found a strong correlation between satisfaction with the learning activities in a 3DVW and an experience of presence, and suggested that until learners have acquired a sense of presence in a virtual world their ability to learn may be impaired. Avatars used in the learning activity can be personalized to create different categories of patients or hospital staff, and if necessary the instructor’s voice can be altered (morphed).

The implementation of 3DVWs in more courses in the pharmacy program is under consideration, however before doing so there is a need to evaluate the perceptions and experiences of the pharmacy students that have participated in exercises in the 3DVW during the course. As noted by Chow et al., it is important to understand the reasons why users accept or reject a new technology [23]. The technology acceptance model (TAM) is one of the most extensively used models to predict and explain the acceptance of a new technology [23]. In this theoretical model it is hypothesized that an individual’s intention to use a technology is controlled by two attitudes: how easy it is to use and its usefulness [23]. Hence, the aim of this study was to explore pharmacy students’ perceptions and experiences of 3DVWs as an instructional tool for clinical pharmacy teaching.

### 1.2. Course Description

The course in clinical pharmacy, 7.5 ECTS was developed in 2011 when the two-year supplementary distance Master’s degree was first offered at Umeå University. The course is mainly web-based but the students also spend three days on campus at the beginning of the course. The course uses digital technologies such as Ping Pong (the program learning management system, LMS), Adobe Connect (a web-based communication program), and a 3DVW for online communication, combined with net based meetings with lecturers at the university. All these digital technologies have been used since the program’s inception in 2011. The course covers various methods for performing clinical pharmacy work and is built on the basis of problem base learning exercises (PBL) such as patient cases containing complex drug related problems (DRPs) that the students work with and discuss. The students need to search for and critically evaluate the literature and apply evidence-based medicine to be able to perform a comprehensive medication review for each individual patient (case). The students present some of the patient cases in Adobe Connect and some of them on campus. Furthermore, to achieve one of the learning objectives: “to communicate with patients and doctors about individual patients’ drug therapy”, the 3DVW is used for presenting the remaining cases. The seminars in the 3DVW are designed as role-playing simulations. Both the teacher and the students take on the roles of specific characters, the student as a clinical pharmacist and the teacher as a patient or doctor. Before the communication exercise starts, the students are provided with a medication list and a medical record for each patient (case) to be able to prepare for the meetings. The student, in the role as a clinical pharmacist, will then meet the patient in the 3DVW and communicate with her/him about their prescribed medications. The student will then perform a medication review with the information gathered from the patient interview, medication list, and medical record. Then, the doctor and student will meet in a ward round in the 3DVW and communicate regarding the DRPs found. For each exercise, the student has 30 min (excluding preparation time), which gives them enough time to perform their task. The exercises are not graded, except one patient case in 3DVW that is used as an assessment task, performed in the way described above. The second assessment is a traditional written assignment where the students are asked to do a medication review for three patient cases. These assessments are graded by the clinical pharmacist who is responsible for the course.

## 2. Materials and Methods

In order to gain insight into the perceptions and experiences of pharmacy students to the use of 3DVWs such as OpenSimulator, a qualitative approach to data collection was adopted [24]. This study adheres to the consolidated criteria for reporting qualitative studies (COREQ) [25].

### 2.1. Participants and Recruitment

Convenience sampling [26], was used to identify second year Master of Pharmacy students who had participated in communicative exercises in the 3DVW on the clinical pharmacy course. An invitation to participate was posted on the notice board of the program learning management system, where students were invited to contribute to the evaluation. Twelve of the fifteen students enrolled in the course agreed to participate. The average age of the participants was 32.8 years (range 25 to 39), three were male and nine were female. All participants reported having no previous experience of 3DVWs prior to enrollment on the clinical pharmacy course. The respondents received a participant information sheet concerning the purpose of the study prior to participation and gave their written consent for the gathered data to be used in this study. Due to the confidential nature of the information revealed, emphasis was placed on reassuring each respondent that anonymity is guaranteed.

### 2.2. Data Collection

Semi-structured interviews with twelve students were carried out in May 2015 by a member of the Center for Educational Development to elicit the students’ perceptions and experiences of carrying out educational activities in a 3DVW. Based on a review of the literature and discussions within the team an interview schedule with a list of topics was developed. The schedule focused on two main areas: (1) relevant background information such as participants’ previous experience of 3DVWs, general computer skills, and any technical problems encountered; and (2) the participants’ perceptions and experiences of using the 3DVW as a tool for learning. The schedule acted as a guide or prompt sheet to ensure that the same topics were covered during the interviews. However, the interview questions were as non-directive as possible and respondents were able to express their own concerns. This allowed relevant issues to emerge and evolve as the study progressed which were then included in the interview schedule.

A pilot test interview was conducted with a participant resident in Umeå to determine the suitability of the questions. After the test, new topics suggested by the participant were included in the schedule, and interview questions considered difficult to answer were reworded. Since the degree is delivered almost entirely online the participants were geographically widely dispersed. Interviews with the remaining 11 participants were therefore carried out in Tromsö, Norway where students were attending laboratory training. All interviews were approximately 25 min in length (range 15–35) and were digitally recorded and later transcribed for analysis. One student was Norwegian and the remaining 11 were Swedish. The respondents’ contributions were anonymized and stored according to research ethics regulations. Qualitative software QSR NVivo^®^ Version 10 (QSR International, Melbourne, Australia) was used to record, store, and organize the data. Thematic analysis was used to analyze interview data [27]. The interview transcripts were read iteratively by the researcher (CE) to gain an overall sense of the data. The interview data was then read again and coded to produce an initial code list until, in the opinion of the researcher, analysis had reached a theoretical saturation. From this basis the data was then coded selectively in terms of emergent themes related to the aims of the study. Quotations were translated from Swedish to English by author CE, and then re-examined and re-translated by author MG for accuracy.

### 2.3. Ethics

No ethical committee approval was sought prior to beginning this research as it is not mandatory by Swedish law for this type of study. Nonetheless, all respondents were provided with information about the research project and signed a written consent to participate.

## 3. Results

Analysis of the data revealed a variety of factors underlying respondents’ perceptions and experiences of using 3DVWs as an instructional tool for clinical pharmacy teaching. The emergent themes are listed in Table 1 and are described in greater detail below. Firstly, background information concerning the participants’ computer skills, prior experience of 3DVWs, and introduction to the 3DVW is described and secondly, the students’ perceptions and experiences of the 3DVW and learning activity are illustrated.

### 3.1. Background Information

#### 3.1.1. General Computer Skills

The mode of delivery for the Master’s degree is web-based; consequently, students studying the degree program have good general levels of computer literacy. Nonetheless, this does not include the use of games and virtual reality. Some participants also indicated that they did not feel themselves to be fully competent computer users; these participants were also those most negative to using 3DVWs for educational purposes and experienced the most problems with the technology:

“Well, what did I think? A little curious at first and then we had the introduction, one hour, and then we had to test the program ourselves. I do not like computer games. I get headaches from sitting too long in front of the computer. I had trouble moving my avatar around and my experience was, now I’ll be honest, that was a bit unnecessary, having to do it through the OpenSimulator.”

Three of the twelve participants had previously studied at Umeå University and thus had prior experience of web-based education. These three were also among the most positive to the use of 3DVWs and found the technology easy to use:

“It was a good way to learn how to communicate with patients and doctors on the right level. We could talk to each other wherever we were. I had no problem with the voice or sound. I think even people with minimal computer skills could easily cope with this.”

It is important to note that none of the participants had previous experience of 3DVWs. However, several had some experience of computer simulation games such as Sims, World of Warcraft, and Habbo.

#### 3.1.2. Instructions and Guidance Provided

All of the students interviewed were satisfied with both the introduction to the technology and to the task. Particularly the introduction to the 3DVW provided in the data lab was described as very pedagogical and thorough.

“The introduction was good. We got to try a little bit; he showed us how to log in, move around and such. There were also recorded instructions to follow. So it was enough”

Nevertheless, some of the students later had problems with the technology when they returned home and installed the software on their own computers:

“Yes, the introduction went very well indeed. It felt like it was easy, but when I did it myself, it wasn’t the same. Because you know, when [the instructor] was there, he explained, “Do this, do like this”. It felt like it was easy, like I could do it but when I got home, it was a bit difficult. But the actual presentation, it went well, it was good.”

Several participants also remarked that there was too much time between the introduction and the assignment as they had almost forgotten how to use the software. Furthermore, the introduction was held in a data lab where some of the computers were out of date and students mentioned that it would have been better if they had used their own computers and been able to download and install the software during the introduction.

#### 3.1.3. Familiarization with the 3DVW

Despite advice given to test and familiarize themselves with the environment before carrying out the tasks less than half of the students did so. The majority of participants waited until 10–15 min before the agreed assessment time to enter the 3DVW:

“I went in maybe 10 min before the meeting should start so that I could find the room. I did. But otherwise, I didn’t go in and test before the meeting or such... Sometimes it went well, sometimes it went not so well...”

Not surprisingly, the students who familiarized themselves with the environment were also those who were most positive to using 3DVWs for communication training:

“Yes, I went into the world twice because I wasn’t so confident about it, so I had to go in and try it out... I didn’t know so much about it, so I knew that I needed to familiarize myself with where I should be for the meeting. So I went in a couple of times to check.”

Interestingly, the participants who went in and tested the environment prior to carrying out the assignment were also those who had previous experience of computer games. Possibly because they were more aware of the problems that could arise, they did not presume it would work and hence tested the system in advance.

Almost all of the students, however, were well prepared for the assignment itself:

“Prior to the seminar, I sat with my friend... We sat on Skype at home and talked through what he thought and what I felt. Why would I ask that question, why would he ask a different question. So we practiced, we took every illness, and analyzed ‘what’s wrong here?’ So I prepared, tried as much as possible, get as much information from the patient so that I could solve the case.”

### 3.2. Perceptions and Experiences of the Virtual Environment

#### 3.2.1. General Attitudes to the VW

The participants’ perceptions of the virtual environment varied greatly. The majority were positive to using a 3DVW for educational purposes. In particular for distance students 3DVWs provide online learning opportunities for communication that are sufficiently immersive and collaborative in ways that current VLE systems cannot. The students saw the advantage in having an online environment where communication can be practiced without the need to travel to campus:

“With the assignment, you get a feeling for how to talk to the patient, how to talk to a doctor. And it was different and fun! … We need to be able to communicate with patients. For example, if you work in any hospital, then you have to know and understand how to talk to them, with a doctor and patient. I thought it was a great exercise actually.”

Participants also felt that the knowledge they had gained in the 3DVW exercise was transferable to their work situation and that other courses that involve communication would benefit from using 3DVWs.

“Both as a clinical pharmacist and community pharmacist I think I’ll find it useful in conversations with customers. It was very helpful learning how to ask about, for example, medication lists and so on and practicing communication.”

For some participants, however, the 3DVW was a barrier and the exchange did not feel authentic:

“No, not entirely. It was like it was a small computer figure who was talking for me. Of course, I knew I had to go into the role and I did, but it still didn’t feel real. Especially when there was so much hassle at times with the sound.”

#### 3.2.2. Perceived Usefulness

The majority of the students carrying out the tasks in the 3DVW described it as meaningful and considered it to have contributed to their learning. The opportunity to adopt the role of the pharmacist in an authentic situation and to practice communication with patients and doctors was perceived by many participants to be particularly useful:

“The task was very practical and it felt good. You had to act as a pharmacist and use your knowledge in practice, and also you had to act correctly in the profession. So it is a good exercise.”

For the majority of the participants, it felt easy to take on the role of the pharmacist in the 3DVW and to carry out the tasks:

“Yes, actually, it did feel real. When I had my first conversation with the patient in OpenSimulator, my boyfriend sat in the same room and listened. When I finished the exercise, he asked ‘Did you just talk with a patient?’ It must have been a realistic conversation!”

Communication with the doctor and patient in the 3DVW was also experienced as authentic:

“To begin with it felt strange [in the 3DVW] but when I met the patient, it felt much more real. I saw the patient lying there in the bed looking ill and I thought okay, now I have to really go for this and enter the role of the pharmacist. Then it felt authentic.”

#### 3.2.3. Perceived Ease of Use

A third of the participants however felt that learning to use the 3DVW took too much time; time that they would rather have used for their studies:

“Yes, I think [the 3DVW] worked well. Sure, it maybe took a little more time. That’s the drawback, perhaps, that it takes time to learn. First, you have the introduction, to go through and create everything, and then you have to get it installed at home. And if you have problems with it, it is clear that it is very time consuming. That’s probably what is negative. You may not always have as much time left for studies as you’d like.”

Nonetheless, many participants indicated that if the 3DVW was used on more courses then they would feel that the time invested in learning how to use the environment was better spent. Of those who were doubtful, the majority of the participants felt that the technology at present is too complicated and time-consuming to learn; if it were easier to use the virtual environment it would be no problem:

“I think it was because you had to learn to download the program, fix an account, log in, and then learn to move around and such. It felt a bit unnecessary because if you compare with Adobe Connect, it’s just one click and you’re inside the room, you enter your name, and you’re inside. Then you can just talk.”

A few participants were unable to solve problems with their audio and used a telephone to carry out the task. Two tried to connect from work and experienced firewall problems and one had a hardware problem with his/her own computer. For these participants the difficulties they experienced with the technology and the unfamiliar environment were a hindrance to being able to adopt the pharmacist’s role.

Although half of the participants reported that they had no problem at all with the technical environment, others reported more or less serious difficulties. Nevertheless, of those with some difficulties not all were directly negative to using the 3DVW. Even participants with audio problems could see some benefits in using a virtual environment “*if the technology worked*”.

Many participants reported that they experienced some degree of stress caused by the unfamiliarity and technical difficulty of using the 3DVW, particularly in an assessment context:

“It was more that you are not familiar with [3DVWs], if you do not play a lot of games and stuff, then you are not familiar, it becomes a stress factor. That is, just a small threshold. I thought it was a little risky in connection with the examination to have to think about that as well. It was a bit stressful. One should not have it in connection with the examination, I think.”

It was also suggested by many participants that the task could have been carried out more easily in Adobe Connect.

“Therefore, I think maybe, it would probably have been just as well to do it through Adobe Connect. I was so stressed that I was late for my meeting [in the 3DVW]. I should be there at one o’clock and I began logging in at a quarter to, but my avatar got stuck and so I was late. When I got to the room, I was already so stressed ... For me, I saw nothing of the environment. I was just stressed out that I wouldn’t get there and be able to do my assignment. So this thing about, this feeling of being in a pharmacy, or that there was a hospital, I didn’t experience it.”

Using Adobe Connect as an alternative has also been discussed by program management. However, it is felt that the opportunities offered by the 3DVW environment to create an authentic task and provide anonymity for students and teachers outweigh the disadvantages. Some students also expressed that the task felt more meaningful when the identity of the other party was unknown and that they felt less nervous and found it easier to adopt the role of the pharmacist as an avatar in the 3DVW than in a role-play situation in Adobe Connect.

“I think the personal anonymity in OpenSimulator is good. You don’t know who the other person is and they can’t ‘see’ you. You are a bit hidden behind the character you play. In Adobe Connect, you can see each other so I think it’s easier in OpenSimulator not to have to worry so much.”

## 4. Discussion

Students in this study perceived the use of 3DVWs in clinical pharmacy education to be a meaningful activity and were in general positive about its use. As mentioned by Cain et al. [28], 3DVWs in pharmacy education offer opportunities for engaged and active learning. Coffman and Klinger [29] described how 3DVWs utilize a “constructivist approach” to learning where students are provided with learning opportunities that challenge them to “learn by experiencing and through applied activities, rather than by direct instruction and passive involvement.” However, healthcare educators need to explore how students engage with these 3DVWs. Consistent with the technology acceptance model (TAM), this study showed how students value the learning experience but the level of engagement relates to not only their computer skills and ease of use but also to the value they place on VWs as a learning environment. In this study, students who described confidence in using computers perceived the 3DVW as easy to use and useful—the ones who stated that they “weren’t computer people” were more negative, did not train (or practice in the 3DVW ) and had greater difficulties; for them technology was seen as a barrier to learning.

However, it is not as simple as thinking that improving students’ general computer skills will improve their learning experience in the 3DVW. Both perceived ease of use and usefulness appear to play an important role for the students. If they do not perceive learning the technology as useful (since it is only used in one course) they do not feel motivated to put the time into learning how to use it. If they also perceive it to be difficult to use, their motivation is even lower. Prosser [30] also discusses the importance of the students’ approaches to learning for acceptance of new technologies in the learning environment. Student learning outcomes from the use of new technologies depend on how they see the aims of the new technologies, how they experience the workload associated with use of technology and their perceptions of what will be rewarded in the assessment. Furthermore, success will depend on whether they approach the technology with the intention to learn and understand or with the intention of just completing the task; that is to say if they have a deep approach to learning or a surface approach [31].

One implication is that the usefulness of 3DVWs needs to be emphasized prior to the activity; it needs to be embedded in the program from year one (used on more courses) and made simpler to use. This may improve student perceptions of learning in 3DVWs for those studying on the Bachelor of Science in Pharmacy program at Umeå University, however only 3 of the 12 respondents had formerly completed this program at Umeå before enrolling on the Master of Pharmacy program. 3DVWs do add an additional layer of complexity to the learning environment. Not only for students but also for teaching staff, and it has been argued that they place new demands on teachers in the design of appropriate activities and the adoption of a student-centered approach to teaching and learning [32,33]. As described by Cain et al. [28], the use of 3DVW applications may be limited by the learning curve associated with their use (both for teachers and students). However, with the advances of technology this barrier could soon be overcome. Current developments within the field of virtual reality and head-mounted displays such as Oculus Rift are certainly promising, however at present this would not be feasible due to the cost of equipping students with the necessary technology. Nevertheless, it is important to consider the cost of upgrading and modernizing the current 3DVW on an ongoing basis and having access to information technology (IT) support. Some changes in the 3DVW activity have been implemented as a response to the results from this study. Instructional videos on how to install and use OpenSimulator are now available on Ping Pong (the program LMS). The students can watch this video any time. A meeting is also now held via Adobe connect (a communication software) where the students meet the person in charge of providing IT support. After this meeting on the same afternoon, the first 3DVW exercise takes place and the rest of the exercises are now held closer to each other (i.e., almost every week).

## 5. Conclusions

3DVWs have an enormous potential for pharmacy education, but they need to be easier to use and well integrated into the curriculum if their advantages are to be exploited. It seems necessary to emphasize to students how 3DVWs can support their learning and also the necessity of spending time familiarizing themselves with the 3DVW environment if they are to have a better learning experience.

## Figures and Tables

**Table 1:** Themes and emergent sub-themes.

Theme	Subthemes
Background information	General computer skills Instruction and guidance provided Familiarization with the 3DVW
Perceptions and experiences of the virtual environment	General attitudes to the 3DVW Perceived usefulness Perceived ease of use
